# Impact of Acute Changes of Left Ventricular Contractility on the Transvalvular Impedance: Validation Study by Pressure-Volume Loop Analysis in Healthy Pigs

**DOI:** 10.1371/journal.pone.0080591

**Published:** 2013-11-19

**Authors:** Vincenzo Lionetti, Simone Lorenzo Romano, Giacomo Bianchi, Fabio Bernini, Anar Dushpanova, Giuseppe Mascia, Martina Nesti, Franco Di Gregorio, Alberto Barbetta, Luigi Padeletti

**Affiliations:** 1 Laboratory of Medical Science, Institute of Life Sciences, Scuola Superiore Sant’Anna, Pisa, Italy; 2 Fondazione CNR/Regione Toscana “G. Monasterio”, Pisa, Italy; 3 Department of Medical and Surgical Critical Care, University of Florence, Florence, Italy; 4 Clinical Research Unit, Medico Spa, Ruban. Padua, Italy; S.G.Battista Hospital, Italy

## Abstract

**Background:**

The real-time and continuous assessment of left ventricular (LV) myocardial contractility through an implanted device is a clinically relevant goal. Transvalvular impedance (TVI) is an impedentiometric signal detected in the right cardiac chambers that changes during stroke volume fluctuations in patients. However, the relationship between TVI signals and LV contractility has not been proven. We investigated whether TVI signals predict changes of LV inotropic state during clinically relevant loading and inotropic conditions in swine normal heart.

**Methods:**

The assessment of RVTVI signals was performed in anesthetized adult healthy anesthetized pigs (n = 6) instrumented for measurement of aortic and LV pressure, dP/dt_max_ and LV volumes. Myocardial contractility was assessed with the slope (Ees) of the LV end systolic pressure-volume relationship. Effective arterial elastance (Ea) and stroke work (SW) were determined from the LV pressure-volume loops. Pigs were studied at rest (baseline), after transient mechanical preload reduction and afterload increase, after 10-min of low dose dobutamine infusion (LDDS, 10 ug/kg/min, i.v), and esmolol administration (ESMO, bolus of 500 µg and continuous infusion of 100 µg·kg−1·min−1).

**Results:**

We detected a significant relationship between ESTVI and dP/dt*max* during LDDS and ESMO administration. In addition, the fluctuations of ESTVI were significantly related to changes of the Ees during afterload increase, LDDS and ESMO infusion.

**Conclusions:**

ESTVI signal detected in right cardiac chamber is significantly affected by acute changes in cardiac mechanical activity and is able to predict acute changes of LV inotropic state in normal heart.

## Introduction

The ideal index of ventricular performance, which describes the pumping properties of the left ventricle, should be sensitive to changes of left ventricular (LV) inotropic state, independent of loading conditions, easily reproducible and safe [1]. Left ventricular pressure-volume (PV) loops are considered to be the gold standard for complete hemodynamic assessment during each cardiac cycle [2]–[3] and are widely used in research to evaluate the LV inotropic state and to quantify mechanical ventricular dyssynchrony in large animal model [4]–[5] and humans [6]–[7]. However, the PV loops assessment by conductance catheter is an invasive and complex approach, and its clinical application does not enable a permanent monitoring of ventricular contractility in patients with pacemaker implants.

In fact, the continuous real-time assessment of the LV myocardial contractility by an implanted device could provide useful information to calibrate the electrical stimulation in proportion to the real inotropic state of the heart and to monitor maladaptive hemodynamic response to electrical therapy of the heart [8] and the overall patient care [9]. Permanent pacemakers and defibrillators could be equipped with haemodynamic sensors suitable for diagnostic applications as well as for the autoregulation of the device itself [10]. At present, the haemodynamic guidance has been proposed in the adaptation of the pacing rate to the metabolic demand [11]–[15] and in atrio-ventricular (AV) and interventricular (VV) delay setting [16]–[18].

The signal of intracardiac impedance is a well known parameter for assessing in real time the acute changes of LV performance of failing heart at rest [19] and during adrenergic stress [17], [20]. Pacing leads bearing just standard electrodes can be used to assess cardiac impedance [21]. Different electrode arrangements are proposed for impedance assessment [17], [21]–[24], namely the unipolar configuration (current is applied between the ventricular tip electrode and the device case and the corresponding voltage drop is measured at the same spots), the bipolar configuration (current is applied between two cardiac electrodes and the corresponding voltage drop is measured at the same spots), or different multipolar configurations (current is applied between two cardiac electrodes and the voltage drop is measured on another electrode pair). The transvalvular impedance (TVI) is a particular example of bipolar configuration, where the atrial pole is represented by the ring electrode placed in right atrium and the ventricular pole by either the tip or ring electrode of the right ventricular lead. The timing of rise and decrease during the cardiac cycle suggests that TVI fluctuation reflects opposite changes in ventricular volume, as TVI increases in the ejection phase and decreases during ventricular filling [15], [25]–[27]. Since it is known the relationship between changes in right ventricular TVI and LV stroke volume (SV) during electrical and pharmacological stimulation in patients [25], it is unknown whether the RV TVI depends upon changes of LV inotropic state. In our study, we have analysed different TVI signals and performed a validation study of the end-systolic TVI (ESTVI) as index of LV contractility. For this purpose, we analysed ESTVI and simultaneous PV loops obtained by conductance catheter during different acute loading and inotropic conditions in healthy pigs.

## Materials and Methods

### Animal handling and instrumentation

Six healthy sexually mature male farm pigs (35±2 kg, body weight), fasted overnight, were sedated with a cocktail of tiletamine hydrochloride and zolazepam hydrochloride (8 mg/kg i.m.) and

premedicated with atropine sulfate (0.1 mg/kg). General anesthesia was subsequently induced with propofol (2–4 mg/kg) and maintained with 1% isoflurane in 60% air and 40% oxygen. Mechanical ventilation was adjusted based on arterial blood gas values [28]. Body temperature was maintained at 36.5°–39°C. Arterial pressure was measured via a fluid filled catheter inserted trough the right carotid artery and attached to a P23ID strain-gauge transducer; although, LV pressure, the maximum and minimum of the first derivative of LV pressure (dP/dt_max_ and dP/dt_min_) and LV volume were measured using a pressure-volume conductance catheter (Millar Instruments Inc, Houston TX, USA) percutaneously inserted through the femoral artery and carefully advanced into the LV cavity under fluoroscopic guidance [29]. A 8F Fogarty large balloon occlusion catheter (Edwards Lifesciences, USA) was advanced into the inferior vena cava (IVC) through a right femoral venotomy; although, an additional large balloon catheter was advanced into the descending thoracic aorta trough the left carotid artery [29]. TVI was measured by standard leads for permanent pacing (Medico 366 and 400, Medico Spa, Padova, Italy) inserted trough the right external and internal jugular vein and advanced in right atrium and ventricular apex under fluoroscopic guidance. While the J-shaped atrial lead was positioned in the right appendage, the RV lead was positioned in the mid-low septum, as previously described in patients [25]–[27].

### Hemodynamic measurements

The hemodynamic parameters were determined during one respiratory cycle and comprised the heart rate (HR), the mean arterial pressure (MAP), LV end-diastolic (EDV) and end-systolic volume (ESV), LV end-diatsolic (EDP) and end-systolic pressure (ESP), the maximum derivative of change in pressure rise over time (dP/dt_max_), the maximum derivative of change in pressure fall over time (dP/dt_min_). The stroke volume (SV) was calculated as the difference between EDV and ESV. LV DP/dt_max_ is an index of the isovolumetric phase of the contraction, which is sensitive of preload, but not of afterload [30]. Conversely, the slope of the end-systolic pressure-volume relationship (ESPVR) (Ees) of the left ventricle was calculated using the first 7–10 beats during brief IVC occlusion [5]. Ees is an ejection phase measure of LV contractility, which is minimally affected by preload and afterload [31]. In addition, we measured the LV contractile state by calculation of LVESP/LVESV at each beat [1]. Effective arterial elastance (Ea) was calculated as an index of LV afterload [32]. The measurement of the area of the LV pressure-volume loop during a cardiac cycle was used as an index of stroke work (SW) [33].

The load-dependent and independent LV performance was evaluated simultaneously to the TVI measurement during each experimental condition. All hemodynamic signals were recorded on an eight-channel Gould polygraph recorder (model 5900; Gould Inc., Cleveland, OH, USA). The analogic signals were recorded through an analogic-digital interface (National Instruments), at a sampling rate of 250 Hz [4]. Digitized data were analysed off-line by custom-made software. Pressure–volume data were analysed off-line by a single observer. All conductance volumes were corrected for parallel conductance and the gain constant Î±.

### TVI measurements

The atrial and the ventricular pacing leads were connected in parallel with an external dual-chamber stimulator (PSA 490, Medico Spa, Padova, Italy) and with the custom-made TVI recorder. TVI was derived between the atrial ring and ventricular tip electrodes, applying subthreshold current pulses of 40 µA at 4 KHz. The waveform was sampled and stored at 1 KHz, together with the atrial and ventricular electrograms (AEGM, VEGM), one surface ECG lead, and one accessory signal (either LVP or LVV) derived as analog output of the PV recording equipment. All tracings were displayed in real-time and then analyzed off-line using commercial software (*Acq*Knowledge, Microsoft, USA). The VEGM signal represented the time marker of electrical ventricular activation and the trigger of TVI measurements. TVI increased in systole and decreased in diastole. The minimum and maximum values recorded in 500 ms after VEGM detection were considered, respectively, as the end-diastolic (ED) and ES TVI. The peak-to-peak TVI amplitude (pkpkTVI) was calculated as the difference between ESTVI and EDTVI. The ratio of the maximum TVI increase in 100 ms to the pkpkTVI in each cardiac cycle (TVI fractional change in 100 ms: TVIfc) represented an index of TVI rate of rise.

### Experimental Protocol

The experiments were conducted in anesthetized and hemodynamically stable animals. We first simultaneously measured hemodynamic and TVI parameters at rest. We accurately evaluated the feasibility of conductance catheter-derived P-V loops and TVI waveform during transient reduction of preload, acute increase of LV afterload, during low dose dobutamine stress (LDDS) and following esmolol infusion. Acute reduction of LV preload was induced by transient occlusion of the IVC via inflation of large balloon [4] in order to produce a 20% drop in systolic blood pressure [34]. Conversely, the brief inflation of the intra-aortic balloon afterwards induced a transient increase of LV afterload [34]. The LV function and TVI signals were afterwards assessed during transient inotropic stimulus by low dose dobutamine stress (LDDS, 10μg/kg/min i.v. for 10 minutes), as previously described [35]. LDDS is a well-established test used to provide a quantitative assessment of the LV contractile reserve in both large animal models [28], [35] and patients [36]–[37].

After a 10-minute washout period, the TVI and hemodynamic parameters were evaluated during transient beta-adrenergic blockade induced by esmolol (bolus of 500μg and continuous infusion of 100 µg·kg^−1^·min^−1^ ), a cardioselective beta1 receptor blocker [38]. Basal measurements were repeated before any test in order to update all reference values. Once the experimental protocol was completed, the anesthetized pigs were euthanized with an intravenous injection of saturated KCl solution. Animal instrumentation and experimental protocol were approved by the Animal Care Committee of the Italian Ministry of Health and was in accordance with the Italian law (DL-116, Jan. 27, 1992), which is in compliance with the National Institutes of Health publication *Guide for the Care and Use of Laboratory Animals*.

### Statistical Analysis

All data are mean values ± standard error of the mean. SPSS 11 for Windows (SPSS Inc, Chicago, IL, USA) was utilized for statistical analysis. Intragroup comparisons were performed using the one way analysis of variance followed by the Bonferroni post-hoc pairwise multiple comparisons.

The changes of ESTVI, LVSV, dP/dt_max_ and LVESP/LVESV were calculated as the ratio between each parameter at baseline and during hemodynamic test. Correlations between groups of values were evaluated calculating the best fit, based on least-squares regression analysis. A good correlation was accepted at R value of ≥0.6. For all the statistical analyses, significance was accepted at P value of <0.05.

## Results

### Hemodynamic and P-V loop analysis

**Left ventricular function during changes in loading conditions.** As shown in Table 1, MAP, LVESP, LVEDP, LVdP/dt_max_, LVdP/dt_min_, LVESV, LVEDV were significantly decreased during preload reduction in the presence of unchanged heart rate and reduced LVSV by 37.5±1.86% (p<0.05) compared to baseline (16±1.6 *vs* 24.5±1.5 ml). The LVESP/LVESV and LVSW were respectively reduced by 10.8±7.1 (p<0.05) and 55±15% (p<0.01) compared to normal loading conditions. Conversely, MAP, LVESP, LVdP/dt_max_, LVdP/dt_min_ and LVESP/LVESV were significantly increased during afterload increase in the presence of reduced LVSV by 54.16±2.8% (p<0.01) compared to baseline (10.5±2 *vs* 24.5±1.5 ml) (Table 1). The LV Ees and Ea were respectively increased during afterload increase by 389.8±29.5 (p<0.00001) and 384.2±18.6 (p<0.001) compared to condition of reduced LV preload (Table 1).

**Table 1:** Absolute values and percentage changes compared with respective baseline values during changes in loading conditions.

	Baseline	Acute Reduction of LV Preload	Acute Increase of LV Afterload
*TVI Parameters*			
ESTVI (Ohm)	822±61.19	867.8±60.14*	844.2±77.36*
EDTVI (Ohm)	691.5±61.18	796±57.5*	755±74*
pk-pk TVI (Ohm)	130.15±8.59	71±8.9*	89±13.16*
*Hemodynamic Parameters*			
HR (bpm)	76.3±4.64	75±5.02	72±4.85
MAP (mmHg)	73.42±15.27	62±13.2*	138±17*#
*P-V Loop Measures and Calculations*			
LVESP (mmHg)	102.57±4.6	67±7.3*	185.5±3.98*#
LVEDP (mmHg)	7.04±1.46	2.48±0.87*	12.57±3.6#
LVdP/dt_max_ (mmHg/s)	1763.1±36.87	1259±64.98*	1990.5± 40.5*#
LVdP/dt_min_ (mmHg/s)	–1029.2±113.3	–733.53±169.11*	–1775±136.2*#
LVESV (ml)	48±1.98	35.3±1.16*	58±2.55*#
LVEDV (ml)	72±2.9	51.3±1.66*	69±3.6#
LVSW (mmHg*ml)	2293±231.95	1032±220.02*	1892.08±245.17#
LVESP/LVESV (mmHg/ml)	2.13±0.13	1.91±0.08	3.2±0.1*#
Ea (mmHg/ml)	4.25±1.1	4.4±1.1	20.6±3.2#
Ees (mmHg/ml)		2±0.13	12±0.6#
*Percentage changes*			
% change to baseline of ESTVI		5.5±1.09	2.7±3
% change to baseline of EDTVI		15.11±2.85	9.2±1.7
% change to baseline of pk-pk TVI		–46±20.9	–29±18.75
% change to baseline of LVdP/dt_max_		–28.58±14.7	12.9±1.18
% change to baseline of LVdP/dt_min_		–28.7±28.4	72.47±21.2
% change to baseline of LVSW		–55±15	–17.48±1.3
% change to baseline of LVESP/LVESV		–10.8±7.1	50.2±2.48
% change to acute reduction of LV preload of Ea		31.29±3–4	384.2±18.6
% change to acute reduction of LV preload of Ees			389.8±29.5

Mean values±S.E.M. n = 6. ESTVI, end-systolic TVI; EDTVI, end-diastolic TVI; pk-pk TVI, peak to peak TVI; HR, heart rate; MAP, mean arterial pressare; LVESP, left ventricular end-systolic pressure; LVEDP, left ventricular end-diastolic pressure; LVESV, left ventricular end-systolic volume; LVEDV, left ventricular end-diastolic volume; LVSW, left ventricular stroke work; Ea, arterial elastance; Ees, end-systolic elastance. * P<0.05 vs baseline; #P<0.05 vs acute preload reduction.

**Left ventricular function during changes in inotropic state.** As shown in Table 2, HR, MAP, LVESP, LVdP/dt_max_, LVdP/dt_min_, LVESP/LVESV and LVSW were significantly increased during LDDS in the presence of increased LVSV by 41.6±1.76% (p<0.05) compared to baseline (35±1.8 *vs* 24±1.5 ml). Similarly, the LVEes was increased by 157±4.5 (p<0.001) compared to baseline. Conversely, the administration of esmolol significantly reduced HR, LVESP, LVdP/dt_max_, LVdP/dt_min_, LVESP/LVESV and LVSW in the presence of reduced LVSV by 40±2.86% compared to baseline (14±2.4 *vs* 24±1.5 ml) (p<0.05) (Table 2). The LVEes was significantly reduced by 38±1% compared to resting condition (baseline).

**Table 2:** Absolute values and percentage changes in contractile indices compared with respective baseline values during inotropic modulation.

	Baseline	LDDS	Esmolol
*TVI Parameters*			
ESTVI (Ohm)	822±61.19	804.6±59.9*	723.8±60.5*#
EDTVI (Ohm)	691.5±61.18	688.3±67.2*	605.9±58.66*#
pk-pk TVI (Ohm)	130.15±8.59	116.3±12.3	117±17.37
*Hemodynamic Parameters*			
HR (bpm)	76.3±4.64	108.2±11.2*	62.5±3.8*#
MAP (mmHg)	73.42±15.27	104.6±3.83*	70.5±11.2#
*P-V Loop Measures and Calculations*			
LVESP (mmHg)	102.57±4.6	128.95±4.4*	83±4.4*#
LVEDP (mmHg)	7.04±1.46	9.3±1.81	10.3±3.6
LVdP/dt_max_ (mmHg/s)	1763.1±36.87	4234.12±268.64*	959.76±80.4*#
LVdP/dt_min_ (mmHg/s)	–1029.2±113.3	–1606.3±126.9*	–580.3±86.3*#
LVESV (ml)	48±1.98	39±2.94*	68.8±3.02*#
LVEDV (ml)	72±2.9	73±1.62	83.2±4.22*
LVSW (mmHg*ml)	2293±231.95	4068±14.25*	1051±67.42*#
LVESP/LVESV (mmHg/ml)	2.13±0.13	3.3±0.13*	1.18±0.15*#
Ea (mmHg/ml)	4.25±1.1	3.79±1.22	5.53±1.2#
Ees (mmHg/ml)		5.14±0.52	1.24±0.16*#
*Percentage changes*			
% change to baseline of ESTVI		–2.23±0.9	–12.6±2.88
% change to baseline of EDTVI		–0.43±2.17	–12.3±4.5
% change to baseline of pk-pk TVI		–10±15	–10±6.3
% change to baseline of LVdP/dt_max_		140.15±8.14	–45.6±3.87
% change to baselien of LVdP/dt_min_		56.07±5.46	–43.63±5.26
% change to baseline of LVSW		77.38±4.87	–54.15±6.5
% change to baseline of LVESP/LVESV		54.8±2.65	–31.4±6
% change to baseline of Ea		10.82±1.1	14.3±6.8
% change to baseline of Ees			–41±7.6

Mean values±S.E.M. n = 6. ESTVI, end-systolic TVI; EDTVI, end-diastolic TVI; pk-pk TVI, peak to peak TVI; HR, heart rate; MAP, mean arterial pressare; LVESP, left ventricular end-systolic pressure; LVEDP, left ventricular end-diastolic pressure; LVESV, left ventricular end-systolic volume; LVEDV, left ventricular end-diastolic volume; LVSW, left ventricular stroke work; Ea, arterial elastance; Ees, end-systolic elastance. * P<0.05 vs baseline; #P<0.05 vs LDDS.

### TVI signals during changes in loading and inotropic conditions

**Figure 1:**
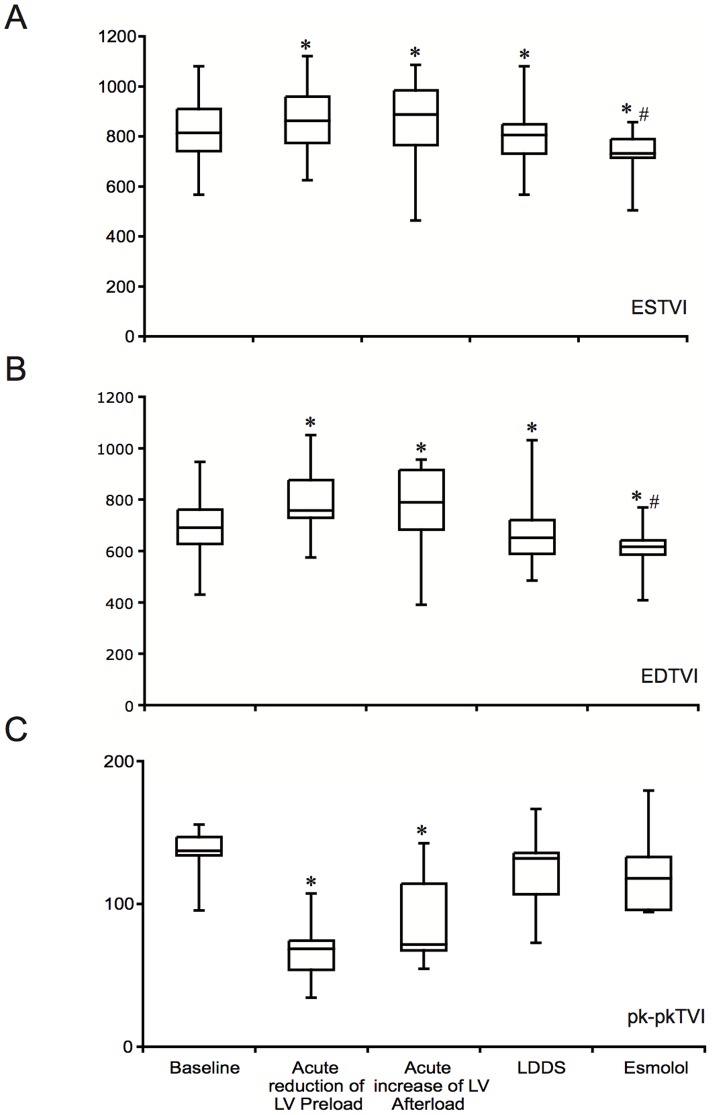
ESTVI (A), EDTVI (B) and pk-pk TVI (C) changes during different loading and inotropic conditions. * *p*<0.05 vs baseline; # *p*<0.05 vs LDDS.

As shown in Table 1 and Figure 1, the acute reduction of LV preload at rest induced a significant increase of ES and EDTVI signals compared to baseline, and a significant reduction of the pk-pk TVI. The above TVI signals reached baseline values following complete IVC balloon deflation (data not shown). Conversely, the transient increase of the LV afterload caused a significant increase of EDTVI in the presence of unchanged ESTVI compared to baseline, yet the pk-pk TVI was reduced by 29±18.75% (Figure 1). As shown in Table 2 and Figure 1, the ESTVI was significantly reduced by 2.23±0.9% during LDDS in the presence of small reduction of EDTVI, and also the pk-pk TVI was reduced.

The abovementioned TVI values during were reduced compared to baseline following esmolol administration, yet pk-pk TVI was unchanged (Table 2 and Figure 1).

### Relationship between RVTVI signals and LV contractility

**Figure 2:**
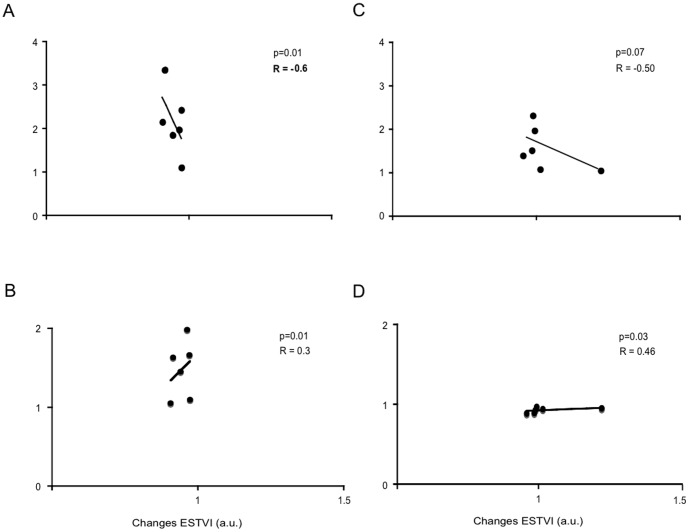
RV ESTVI-LV contractility relationship during changes in different loading conditions. Correlations between changes in RV end systolic TVI (ESTVI) and in left ventricular (LV) stroke volume (SV) or maximum of the first derivative of LV pressure (dP/dt_max_) during preload reduction (A,B) and increase of LV afterload (C, D). Changes normalized to baseline values.

**Figure 3:**
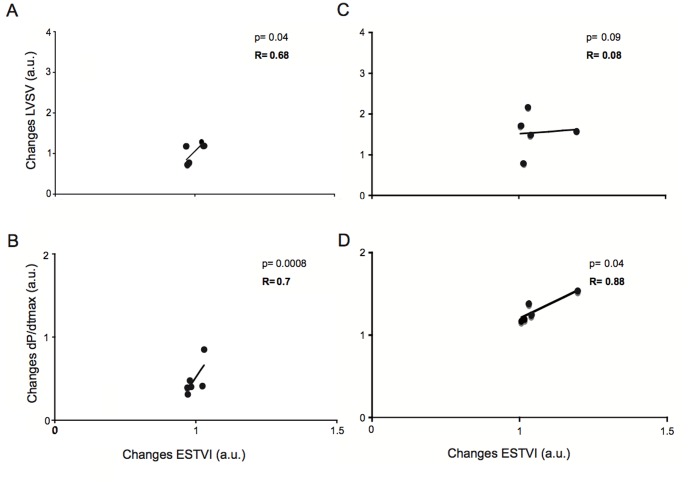
RV ESTVI-LV contractility relationship during changes in different inotropic conditions. Correlations between changes in RV end systolic TVI (ESTVI) and in left ventricular (LV) stroke volume (SV) or in maximum of the first derivative of LV pressure (dP/dt_max_) during low dose dobutamine stress (LDDS) (A,B) and following esmolol infusion (C, D). Changes normalized to baseline values.

**Figure 4:**
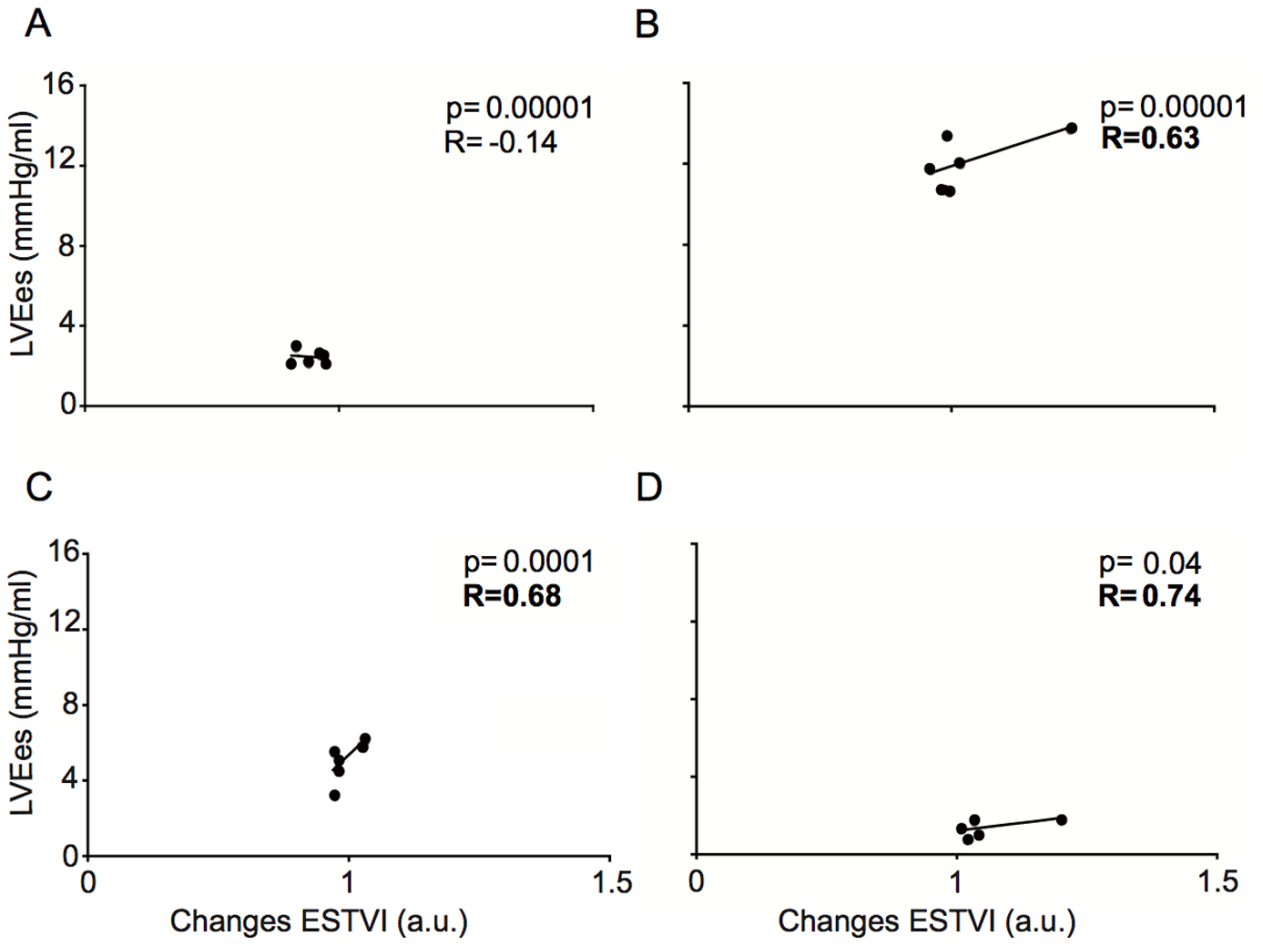
RV ESTVI- LV Ees relationship during changes in different loading and inotropic conditions. Correlation between changes in RV end-systolic TVI (ESTVI) and left ventricular Ees during preload reduction (A), increase of afterload (B), low dose dobutamine stress (C) and following esmolol infusion (D). Changes normalized to baseline values.

As shown in Figure 2A, ESTVI was significantly and inversely related to LVSV during preload reduction. The direct relationship between ESTVI and LVdP/dt_max_ during preload reduction was significant and weak (Figure 2B). Conversely, ESTVI was directly and significantly related to dP/dt_max_ (Figure 2D) during LV afterload increase. As shown in Figure 3, we found a direct and significant correlation between changes in ESTVI and LVSV (Figure 3A) or dP/dt_max_ (Figure 3B) in response to LDDS. In addition, there was a direct a significant correlation between changes in ESTVI and dP/dt_max_ following esmolol infusion (Figure 3D). As shown in Figure 4B, C and D, we found a significant and direct correlation between ESTVI and LVEes during increasing of LV afterload, LDDS and esmolol infusion. No significant correlations were found between the abovementioned hemodynamic parameters and EDTVI or pk-pk TVI (data not shown).

## Discussion

The continuous and minimally invasive real-time assessment of the myocardial contractility is a clinically relevant issue, mainly in patients receiving a pacemaker-induced electrical stimulation of the heart. The implantable haemodynamic sensors, currently proposed for clinical use, are generally aimed at recording intracardiac pressure or other indirect markers of the cardiac contraction strength that have been correlated with the LV dP/dt _max_ [39], [40]. However, dP/dt _max_ is an index of the isovolumetric phase of the LV contractile function, which is more sensitive of preload and does not reflect the myocardial inotropism of the left ventricle [30]. Conversely, the slope of the end-systolic pressure-volume relationship (ESPVR) should be determined to measure the mechanical performance of the myocardium in a load-independent fashion [31–1] and to assess the properties of innovative inotropic sensors, such as TVI, under different hemodynamic conditions.

In our experimental study, preload changes were induced by IVC occlusion that reduced venous return, RV filling and pulmonary output. A secondary decrease of LVSV, LVdP/dt_max_, LVESP/LVESV and LVSW was clearly evident within 2 cycles after beginning balloon inflation. In this experimental condition, both EDTVI and ESTVI were increased, and pk-pkTVI was reduced (Figure 1). In the presence of preload reduction, we found that ESTVI significantly predicts the changes of LVSV (Figure 2A). Our experimental findings confirmed the inverse relationship between RVTVI and RV volume [15], [25]–[27]. In fact, TVI increased whenever the RV volume was supposed to decrease, providing correct information on relative changes in RV preload and stroke volume. However, ESTVI was not so sensitive to predict changes of LVdP/dt_max_ (Figure 2B) and Ees (Figure 4A) at that magnitude of preload reduction.

After recovering the baseline hemodynamic values, an acute increase of LV afterload was obtained by partial occlusion of the thoracic aorta, which entailed a prompt rise in LV pressures and Ees. The LVSV was decreased and LVESV was increased compared to baseline. The acute reduction of the LV output caused a transient reduction of RV filling, with associated reduction in the pulmonary SV, which at the steady state must be equal to LVSV. In agreement with this model, the EDTVI and ESTVI were increased and pk-pkTVI was decreased (Figure 1). However, the ESTVI did not predict transient reduction of LVSV (Figure 2C) and LVdP/dt_max_ (Figure 2D) in the presence of increased isovolumetric contractility. Conversely, we found that ESTVI accurately predicts the increase of LV Ees during acute increase of LV afterload (Figure 4B).

Changes in RV preload were generally coupled with corresponding LV SV modifications. However, the observed increase of ESTVI during rise of LV afterload probably was mostly related to the reduction of RV preload. Knowledge of TVI changes under different hemodynamic conditions might allow a comprehensive interpretation of simultaneous modifications in the contraction strength. It could be essential in the regulation of the AV delay in a dual-chamber stimulator, which usually aims at the optimal ventricular filling (i.e.: the highest possible preload).

The direct relationship found between ESTVI and LVEes during inflation of the aortic balloon suggested ESTVI as potential index of LV inotropic state. For this purpose, we performed the abovementioned measurements during selective inotropic conditions.

Low dose dobutamine stimulation induced a marked positive inotropic response characterized by the expected significant increase of LV pressures, stroke work and LVEes. In spite of the clear-cut effects on pressure-related parameters, the LVSV was not significantly increased compared to baseline, probably due to the high cardiac rate and consequent shortening of the total filling time. We observed, in fact, a small reduction of the ESTVI and EDTVI in the presence of unchanged pk-pk TVI compared to baseline condition. However, we observed that ESTVI accurately predicts changes of LV contractility (Figures 3B) during positive inotropic stimulus even in the absence of significant preload changes. Our experimental data confirmed previous study demonstrating that dobutamine markedly increases RV contractility and intracardiac impedance [39]. In order to better validate the inotropy-sensing of the ESTVI, we repeated the TVI and PV loops assessment following the administration of esmolol, a selective blocker of cardiac beta-adrenergic receptors type 1. Esmolol induced a large reduction of LV dP/dt _max_, SW ad Ees in the presence of marked reduction of LVSV. Surprisingly, we observed a significant reduction of ESTVI and EDTVI in the presence of unchanged pk-pk TVI. In addition, we found a direct and significant correlation between changes of ESTVI and LV dP/dt_max_ (Figure 3D) or LV Ees (Figure 4D). On the basis of our results, we suppose that the SV-ESTVI inverse relationship was unpredictable in the presence of negative inotropic stimulus (LVEes < 2 mmHg/ml) and reduced preload. Since changes of myocardial electrical activity does not affect myocardial electrical impedance measurements [41], it is conceivable that the changes of ES TVI signals in the presence of blocks of cardiac beta-1 adrenoreceptors mainly reflect changes of myocardial impedance rather than right ventricular blood pool impedance. In our experimental model, the infusion of esmolol could have compromised contractile function to such an extent as to cause the formation of moderate myocardial edema [42], which is expected to reduce the myocardial electrical impedance [43]. This finding supports ESTVI as sensor of myocardial contractile function related to changes of myocardial impedance.

Our findings are encouraging and suggest that ESTVI has good potential for use in the permanent beat-by-beat surveillance of the LV inotropic state in patients with pacemakers. However, there is still much investigation required before ESTVI is considered a reliable sensor of LV inotropic state in the presence of disarrangement of myofilaments, myocardial dyssynchrony or heart failure.

### Limitations of our study

The study was performed in the presence of intrinsic AV conduction. It remains to be established whether the TVI properties reported in this study might be affected by changes in right ventricular pacing.

## Conclusions

Our experimental data demonstrates that TVI can real-time and accurately detect LV preload modifications. In addition, the changes of ESTVI easily predict acute changes of the LV contractile function of normal swine hearts under different inotropic conditions.
